# Comparative effects of different dietary patterns on glycemic control in community-managed patients with type 2 diabetes

**DOI:** 10.1097/MD.0000000000047204

**Published:** 2026-01-23

**Authors:** Yanling Zheng, Haiyan Fan, Wenyong Kong

**Affiliations:** a Department of General Medicine, Ziyueshan Community Health Service Station, Longgang District Second People’s Hospital, Shenzhen City, Guangdong Province, China; b Department of General Medicine, Changkeng Community Health Service Center, Longgang District Second People’s Hospital, Shenzhen City, Guangdong Province, China; c Department of General Medicine, Lucheng Community Health Service Station, Longgang District Second People’s Hospital, Shenzhen City, Guangdong Province, China.

**Keywords:** dietary patterns, fasting plasma glucose, glycemic control, postprandial blood glucose, type 2 diabetes mellitus (T2DM)

## Abstract

Dietary patterns play a crucial role in the management of type 2 diabetes mellitus (T2DM), particularly in community-managed settings where personalized care may be limited. This study aimed to evaluate the comparative effects of different dietary patterns on glycemic control in community-managed T2DM patients. A retrospective evaluation was conducted at our hospital, including 860 community-managed T2DM patients. Dietary patterns were identified through factor analysis, and their association with fasting plasma glucose (FPG) and postprandial 2-hour blood glucose control was assessed. Logistic regression models were adjusted for potential confounding factors, including patient demographics, clinical characteristics, and the scores of the other dietary patterns. Six distinct dietary patterns were identified: animal protein, whole grains–plant protein, nuts–fruits, refined grains–vegetables, dairy, and sugary beverages. Patients in the highest quartile (Q4) of the nuts–fruits dietary pattern had a 49.9% reduced risk of poor FPG control compared to those in the lowest quartile (Q1) (*P* < .001), suggesting a protective effect of this pattern. Conversely, patients in Q4 of the sugary beverages pattern had a 3.710-fold increased risk of poor postprandial glucose control compared to those in Q1 (*P* = .021). These findings indicate that certain dietary patterns significantly influence glycemic control, with high sugar consumption adversely impacting postprandial glucose levels. This study demonstrated that dietary patterns significantly affect glycemic control in community-managed T2DM patients. The nuts–fruits pattern improved FPG control, whereas the sugary beverages pattern worsened postprandial glucose regulation, underscoring the importance of promoting healthy, nutrient-dense diets in diabetes management.

## 1. Introduction

The global prevalence of type 2 diabetes mellitus (T2DM) has reached epidemic proportions, presenting significant public health challenges and necessitating effective management strategies. This condition is characterized by chronic hyperglycemia resulting from insulin resistance and impaired insulin secretion. As T2DM primarily affects individuals in community settings, effective management strategies are crucial for improving patient outcomes.^[1,2]^ Glycemic control is paramount in the management of T2DM, as it significantly reduces the risk of complications such as cardiovascular disease, nephropathy, retinopathy, and neuropathy. The primary goal of treatment is to maintain glycemic levels within target ranges, which is typically achieved through a combination of lifestyle modifications, pharmacotherapy, and regular monitoring. Among these, dietary patterns play a crucial role, as the composition and quality of diet can influence glycemic response and overall metabolic health.^[3-5]^

Research has shown that various dietary patterns, including the Mediterranean diet, low-carbohydrate diet, and plant-based diets, have differing impacts on glycemic control. For instance, the Mediterranean diet, rich in healthy fats, whole grains, fruits, and vegetables, has been associated with improved glycemic control and cardiovascular risk factors.^[6,7]^ Conversely, low-carbohydrate diets may lead to more immediate improvements in glycemic indices but may be challenging to adhere to long-term. Similarly, plant-based diets have gained attention for their potential benefits in glycemic management due to their high fiber content and low glycemic load. Despite the increasing evidence supporting the influence of dietary patterns on glycemic control, there remains a paucity of research specifically comparing these dietary strategies within community-managed settings.^[8,9]^ The diverse dietary habits and cultural considerations in different communities further complicate the evaluation of optimal dietary approaches for T2DM management.

This study aims to bridge this gap by systematically comparing the effects of various dietary patterns on glycemic control in community-managed patients with T2DM. By evaluating the efficacy of different dietary approaches, we hope to identify practical and culturally relevant strategies that can be easily implemented in community settings, ultimately enhancing patient engagement and adherence to dietary recommendations. Furthermore, understanding how these dietary patterns interact with individual patient characteristics may pave the way for more personalized dietary interventions, fostering better management of T2DM.

## 2. Materials and methods

### 2.1. Study design

This study undertook a retrospective evaluation at our hospital to assess the comparative effects of different dietary patterns on glycemic control in community-managed patients with T2DM. We selected all T2DM patients who attended our hospital between January 1, 2021 and June 30, 2024. Dietary patterns and corresponding glycemic control levels were meticulously recorded for each patient. The research methodology, objectives, and protocols were developed in accordance with the STROBE (strengthening the reporting of observational studies in epidemiology) guidelines.^[10]^ A total of 860 eligible patients were ultimately included in the analysis. Informed consent was obtained from all subjects via telephone. The study’s design and protocols were reviewed and approved by the ethics committee of (approval no. 20240709057). All procedures adhered to relevant guidelines and the ethical principles of the Declaration of Helsinki. Participant confidentiality was strictly maintained, with all personal identifiers removed prior to data analysis.

### 2.2. Inclusion and exclusion criteria

Inclusion criteria: diagnosis of T2DM: participants must have a confirmed diagnosis of T2DM according to the American Diabetes Association criteria^[11]^; age range: patients aged 18 years and older were included to ensure a broad representation of adult participants; community management: participants must be managed primarily within the community setting, receiving regular follow-up care from community health services or outpatient clinics; dietary recall: individuals must be able to provide accurate dietary recall and documentation of their dietary patterns over the preceding 3 months; and informed consent: participants must have provided informed consent to participate in the study, allowing for the collection and analysis of their medical and dietary data.

Exclusion criteria – type 1 diabetes mellitus: patients diagnosed with type 1 diabetes or any other forms of diabetes (e.g., gestational diabetes) were excluded from the study to focus specifically on T2DM; severe comorbid conditions: individuals with severe comorbid conditions (e.g., uncontrolled cardiovascular disease, chronic kidney disease stage 4 or 5, or severe liver disease) that may confound glycemic control were excluded; recent dietary interventions: participants who had undergone significant dietary interventions or changes in medication regimen within the last 3 months were excluded to minimize the impact of these factors on glycemic control; and pregnancy or lactation: pregnant or lactating women were excluded due to the potential for altered metabolic responses and dietary needs during these periods.

### 2.3. Data collection

General data collection: Comprehensive demographic data were collected, including sex, age, education level, occupation, household income, residence, duration of T2DM, smoking history, and alcohol consumption. Physical measurements such as height, weight, waist circumference, and blood pressure were taken, with blood pressure readings averaged over 3 separate assessments. Body mass index (BMI) was calculated using the formula: BMI = weight (kg)/height (m^2^).

Dietary information collection: Dietary information was gathered using the Chinese Food Composition Table to classify food items into 14 distinct food groups. These groups included: refined grains (e.g., rice and related products, steamed buns, noodles), whole grains (e.g., corn, buckwheat, millet), tubers (e.g., potatoes, yams, taro, sweet potatoes), fresh vegetables (seasonal consumption considered), fresh fruits (seasonal consumption considered), legumes and their products (e.g., tofu, yuba, dried bean curd), dairy products (e.g., milk, yogurt, cheese), meat (e.g., pork, beef, lamb), poultry (e.g., chicken, duck), seafood (e.g., freshwater fish, ribbonfish, shrimp, crab, snails, clams), eggs (e.g., chicken eggs, duck eggs), nuts (e.g., sunflower seeds, peanuts, walnuts, cashews, pistachios, hazelnuts, pine nuts), sugary beverages (e.g., carbonated drinks, commercial tea drinks, sports drinks), and fruit and vegetable juices. A standardized dietary questionnaire developed by national guidelines was employed to retrospectively assess the frequency and average intake of various food items over the past year.^[12]^ Food intake frequency was converted into daily consumption occurrences, with average intake calculated as follows: average intake per food item = daily consumption frequency × average serving size. Serving sizes were recorded based on raw weight, edible portion weight, and market weight, with solid foods documented in grams (g) and liquid foods in milliliters (mL).

Laboratory assessments: Laboratory testing was conducted to measure fasting plasma glucose (FPG) and 2-hour postprandial plasma glucose following a 100 g standardized steamed bun meal. FPG levels of ≤7.0 mmol/L were considered well-controlled, while postprandial glucose levels of ≤10.0 mmol/L were deemed within the acceptable range for glycemic control.

### 2.4. Dietary pattern construction and factor analysis

To construct dietary patterns, we employed factor analysis to reduce the dimensionality of food types and standardize the intake amounts of all food items. To enhance the interpretative value of each factor, we applied varimax rotation to the initial factor loading matrix. A threshold of factor loading ≥0.400 was established as the retention criterion for food groups within the dietary patterns. The decision regarding the number of dietary patterns to retain was based on a combination of eigenvalues >1, scree plot analysis, the interpretability of the dietary patterns, and their contribution to explained variance. The dietary pattern scores were calculated based on the intake levels of food groups associated with each dietary pattern, weighted by their respective factor loadings. In the overall patient population surveyed, the mean score for each dietary pattern was set to 0, with a standard deviation of 1. Each patient received corresponding scores for various dietary patterns, with positive scores indicating an intake level above the average, while negative scores reflected an intake below the average. The magnitude of these scores represents the degree of alignment between the patient’s dietary intake and the defined dietary patterns.

### 2.5. Statistical analysis

Statistical analyses were performed using SPSS version 27.0 (IBM Corp., Armonk, NY, USA) and R Studio version 4.1.2 (RStudio, PBC, Boston, MA, USA). For normally distributed continuous variables, data are presented as mean ± standard deviation, with comparisons between groups conducted using independent samples *t*-tests. For non-normally distributed continuous variables, data are reported as median (Q1, Q3), and group comparisons were made using the Mann–Whitney *U* test. Categorical variables are expressed as counts (percentages), with differences between groups assessed using the chi-squared (χ²) test. Missing values in household income and expenditure data were addressed using Little’s missing completely at random test, which indicated that the data were missing completely at random. Consequently, missing data were imputed using the expectation–maximization algorithm. Factor analysis was utilized to construct dietary patterns, and binary logistic regression analysis was employed to examine the relationship between dietary pattern scores and levels of FPG as well as postprandial glucose control at 2 hours. A 2-sided *P* value of <.05 was considered statistically significant.

## 3. Results

### 3.1. Clinical baseline characteristics

The clinical baseline characteristics of the 860 community-managed T2DM patients are summarized in Table 1. The median age of the patients was 57.41 years (Q1, Q3: 51.01, 64.10), with 52.5% being male. The majority of patients had an education level of middle school or below (89.4%), and a significant proportion (62.8%) were employed in administrative positions. The prevalence of smoking and alcohol consumption was 30.0% and 27.2%, respectively. Regarding socioeconomic status, the median annual household income was 12,000 CNY (Q1, Q3: 6000, 21,714), while the median annual household expenditure was 10,000 CNY (Q1, Q3: 5000, 16,983). The median duration of T2DM was 7.17 years (Q1, Q3: 3.58, 12.08), reflecting a diverse range of disease progression among the patients. In terms of dietary intake, the patients consumed a median of 300 g (Q1, Q3: 200, 450) of refined grains per day, while whole grains and tubers were consumed in smaller quantities. Fresh vegetable and fruit intake was considerable, with a median of 300 g (Q1, Q3: 200, 500) of vegetables and 42.86 g (Q1, Q3: 8.57, 100.00) of fruits per day. Dairy and meat consumption varied widely, while poultry, seafood, and nuts were consumed in smaller amounts. The median BMI was 25.65 kg/m^2^ (Q1, Q3: 23.53, 27.90), with a mean waist circumference of 89.91 ± 9.18 cm. Blood pressure measurements indicated a median systolic blood pressure of 130.83 mm Hg (Q1, Q3: 120.33, 143.92) and a diastolic blood pressure of 78.67 mm Hg (Q1, Q3: 72.33, 85.00), reflecting the overall cardiovascular risk profile of the cohort (Table 1).

**Table 1:** Clinical baseline characteristics of community-managed type 2 diabetes patients.

Observation index	Total population (860 cases)
Age (yr, M(Q1, Q3))	57.41 (51.01, 64.10)
Male, n (%)	451 (52.5)
Education level, n (%)	
Primary or below	227 (26.4)
Middle school	540 (62.8)
College or above	91 (10.6)
Occupation, n (%)	
Technical positions	138 (16.0)
Administrative positions	540 (62.8)
Service and others	181 (21.0)
Smoking, n (%)	258 (30.0)
Alcohol consumption, n (%)	234 (27.2)
Annual household income (CNY, M(Q1, Q3))	12,000 (6000, 21,714)
Annual hHousehold expenditure (CNY, M(Q1, Q3))	10,000 (5000, 16,983)
T2DM duration (yr, M(Q1, Q3))	7.17 (3.58, 12.08)
Refined grains (g, M(Q1, Q3))	300.00 (200.00, 450.00)
Whole grains (g, M(Q1, Q3))	41.43 (14.29, 90.00)
Tubers (g, M(Q1, Q3))	21.43 (7.14, 50.00)
Legumes and products (g, M(Q1, Q3))	28.57 (14.29, 50.00)
Fresh vegetables (g, M(Q1, Q3))	300.00 (200.00, 500.00)
Fresh fruits (g, M(Q1, Q3))	42.86 (8.57, 100.00)
Dairy and products (g, M(Q1, Q3))	28.57 (0.00, 200.00)
Meat (g, M(Q1, Q3))	21.43 (6.67, 50.00)
Poultry (g, M(Q1, Q3))	1.83 (0.00, 10.00)
Seafood (g, M(Q1, Q3)0	1.67 (0.00, 7.14)
Eggs (g, M(Q1, Q3))	50.00 (28.57, 60.00)
Nuts (g, M(Q1, Q3))	2.86 (0.00, 20.00)
BMI (kg/m^2^, M(Q1, Q3))	25.65 (23.53, 27.90)
Waist circumference (cm, *x̄* ± *s*)	89.91 ± 9.18
Systolic blood pressure (mm Hg, M(Q1, Q3))	130.83 (120.33, 143.92)
Diastolic blood pressure (mm Hg, M(Q1, Q3))	78.67 (72.33, 85.00)

BMI = body mass index, T2DM = type 2 diabetes mellitus.

### 3.2. Dietary pattern construction

In this study, all food groups were included in a factor analysis model to identify distinct dietary patterns among community-managed T2DM patients. The Kaiser–Meyer–Olkin statistic for sampling adequacy was 0.646, and Bartlett’s test of sphericity (χ² = 1125.517, *P* < .001) confirmed that the data was suitable for factor analysis. The factor analysis extracted 6 factors with eigenvalues >1, which together accounted for 56.5% of the total dietary variance (Table 2). The individual contributions of these factors to total variance were 15.4%, 10.0%, 8.3%, 8.1%, 7.6%, and 7.2%, respectively. Collinearity tests were conducted to ensure the independence of the dietary patterns, with results indicating that tolerance values were all >0.1 and the variance inflation factor values were <10, indicating no evidence of multicollinearity. Based on the highest factor loadings of specific food groups in each pattern, the 6 dietary patterns were labeled accordingly.

**Table 2:** Factor loadings of food groups across different dietary patterns in community-managed type 2 diabetes patients.

Food groups	Animal protein	Whole grains–plant protein	Nuts–fruits	Refined grains–vegetables	Dairy	Sugary beverages
Poultry	0.823	0.056	−0.023	−0.043	0.069	0.082
Livestock meat	0.724	0.071	0.079	0.197	0.017	−0.053
Seafood	0.471	0.044	0.371	−0.048	−0.021	−0.011
Whole grains	−0.084	0.717	−0.112	0.071	−0.020	−0.042
Tubers	0.016	0.627	−0.023	0.143	0.164	0.037
Legumes and products	0.167	0.588	0.254	−0.030	−0.130	0.083
Eggs	0.181	0.502	0.106	−0.272	−0.041	−0.046
Nuts	0.064	0.022	0.713	−0.021	0.046	−0.012
Fresh fruits	0.051	0.035	0.719	0.168	0.091	0.035
Refined grains	0.052	0.012	0.074	0.799	−0.261	0.026
Fresh vegetables	0.120	0.083	0.109	0.612	0.441	−0.046
Dairy and products	0.034	−0.007	0.107	−0.090	0.861	0.060
Fruit and vegetable Juice	−0.078	0.122	0.135	−0.074	−0.083	0.741
Sugary beverages	0.099	−0.108	−0.115	0.067	0.134	0.710

The animal protein pattern (factor 1) was characterized by high loadings for livestock meat, poultry, and seafood, reflecting a diet rich in animal-based protein sources. The whole grains–plant protein pattern (factor 2) showed high factor loadings for whole grains, tubers, and legumes and their products, indicating a diet high in plant-based proteins and complex carbohydrates. The nuts–fruits pattern (factor 3) was primarily driven by high factor loadings for nuts and fresh fruits, highlighting a diet rich in healthy fats and vitamins. The refined grains–vegetables pattern (factor 4) was defined by high consumption of refined grains and fresh vegetables, suggesting a mixed carbohydrate-fiber intake. The dairy pattern (factor 5) was dominated by dairy and related products, reflecting a diet rich in calcium and dairy-based protein. Lastly, the sugary beverages pattern (factor 6) showed high loadings for fruit and vegetable juices and sugary beverages, indicating a diet with higher sugar intake through beverages (Table 2).

### 3.3. Association between dietary patterns and fasting plasma glucose control rate

Patients were divided into 4 groups (Q1, Q2, Q3, and Q4) based on the quartiles of their dietary pattern scores. FPG levels were categorized, with FPG ≤ 7.0 mmol/L assigned a value of 0 (adequate control) and FPG > 7.0 mmol/L assigned a value of 1 (poor control). Logistic regression models were used to evaluate the association between dietary patterns and FPG control, adjusting for potential confounding factors, including the scores of the other 4 dietary patterns, age, duration of T2DM, region, education level, occupation, annual per capita household income, annual per capita household expenditure, smoking, alcohol consumption, weekly physical activity, exercise intensity, daily sleep duration, BMI, waist circumference, systolic and diastolic blood pressure. The results indicated that compared to patients in Q1 of the nuts–fruits dietary pattern, those in Q4 had a 49.9% reduced risk of poor FPG control, and this association was statistically significant (*P* < .001, Table 3).

**Table 3:** Binary logistic regression results of 6 dietary patterns and their association with fasting plasma glucose (FPG) control rate.

Dietary patterns	Q1 group	Q2 group	Q3 group	Q4 group	*P* value
Animal protein	≤−0.460	(−0.460, −0.263)	(−0.263, 0.144)	>0.144	–
Model 1	1.000	0.729 (0.527–1.071)	0.781 (0.541–1.221)	0.812 (0.540–1.187)	.375
Model 2	1.000	0.757 (0.496–1.165)	0.871 (0.573–1.320)	0.837 (0.548–1.288)	.679
Model 3	1.000	0.865 (0.542–1.315)	1.033 (0.645–1.531)	1.169 (0.727–1.789)	.679
Whole grains–plant protein	≤−0.599	(−0.599, −0.280)	(−0.280, 0.271)	>0.271	–
Model 1	1.000	0.681 (0.454–1.013)	0.744 (0.493–1.217)	1.041 (0.699–1.498)	.719
Model 2	1.000	0.869 (0.566–1.689)	0.836 (0.572–1.221)	0.853 (0.614–1.162)	.365
Model 3	1.000	1.156 (0.740–1.769)	0.906 (0.576–1.415)	0.762 (0.474–1.198)	.354
Nuts–fruits	≤−0.496	(−0.496, −0.249)	(−0.249, 0.231)	>0.231	–
Model 1	1.000	0.382 (0.255–0.544)	0.527 (0.351–0.763)	0.409 (0.271–0.577)	<.001
Model 2	1.000	0.468 (0.305–0.734)	0.589 (0.380–0.767)	0.487 (0.317–0.593)	<.001
Model 3	1.000	0.493 (0.244–0.546)	0.584 (0.356–0.889)	0.501 (0.256–0.668)	<.001
Refined grains–vegetables	≤−0.726	(−0.726, −0.118)	(−0.118, 0.546)	>0.546	–
Model 1	1.000	0.689 (0.472–1.029)	0.889 (0.603–1.265)	0.811 (0.532–1.198)	.269
Model 2	1.000	0.646 (0.412–1.064)	0.831 (0.563–1.267)	0.771 (0.505–1.087)	.157
Model 3	1.000	0.781 (0.492–1.187)	0.958 (0.633–1.599)	0.859 (0.545–1.319)	.267
Dairy	≤−0.681	(−0.681, −0.242)	(−0.242, 0.636)	>0.636	–
Model 1	1.000	0.922 (0.665–1.328)	0.989 (0.742–1.327)	0.692 (0.452–1.010)	.233
Model 2	1.000	0.829 (0.525–1.268)	0.949 (0.645–1.346)	0.795 (0.511–1.285)	.245
Model 3	1.000	0.841 (0.539–1.270)	1.062 (0.661–1.566)	1.005 (0.516–1.285)	.356
Sugary beverages	≤−0.249	(−0.249, −0.180)	(−0.180, −0.096)	>−0.096	–
Model 1	1.000	0.891 (0.602–1.168)	1.015 (0.693–1.489)	0.961 (0.649–1.396)	.917
Model 2	1.000	0.879 (0.535–1.353)	1.064 (0.721–1.564)	0.862 (0.521–1.567)	.759
Model 3	1.000	0.890 (0.512–1.763)	1.051 (0.621–1.561)	0.871 (0.551–1.354)	.907

Model 1: Unadjusted for confounding factors.

Model 2: Adjusted for other dietary patterns.

Model 3: Adjusted for age, duration of diabetes, region, education level, occupation, annual per capita household income, annual per capita household expenditure, smoking, alcohol consumption, weekly physical activity level, exercise intensity, daily sleep duration, body mass index (BMI), waist circumference, systolic blood pressure, diastolic blood pressure, fasting plasma glucose, glycated hemoglobin (HbA1c), total cholesterol, triglycerides, low-density lipoprotein cholesterol (LDL-C), high-density lipoprotein cholesterol (HDL-C), and serum uric acid.

FPG = fasting plasma glucose, HbA1c = glycated hemoglobin, HDL-C = high-density lipoprotein cholesterol, LDL-C = low-density lipoprotein cholesterol.

### 3.4. Association between dietary patterns and postprandial 2-hour blood glucose control rate

Postprandial 2-hour blood glucose levels were classified into 2 categories, with glucose levels ≤ 10.0 mmol/L considered adequate control (assigned a value of 0) and levels > 10.0 mmol/L considered poor control (assigned a value of 1). Logistic regression models were constructed to assess the association between 6 dietary pattern scores and postprandial blood glucose control. The models adjusted for the scores of the other 4 dietary patterns, as well as key demographic and clinical factors, including age, duration of diabetes, region, education level, occupation, annual per capita household income, annual per capita household expenditure, smoking, alcohol consumption, weekly physical activity, exercise intensity, daily sleep duration, BMI, waist circumference, systolic and diastolic blood pressure. Compared with patients in Q1 of the Sugary Beverages dietary pattern score, those in Q4 had a significantly increased risk of poor postprandial blood glucose control, with an odds ratio of 3.710 (*P* = .021, Table 4).

**Table 4:** Binary logistic regression analysis of the association between 6 dietary pattern scores and postprandial 2-hour blood glucose control rate.

Dietary patterns	Q1 group	Q2 group	Q3 group	Q4 group	*P* value
Animal protein	≤−0.446	(−0.446, −0.261)	(−0.261, 0.141)	>0.141	–
Model 1	1.000	1.142 (0.558–2.470)	0.908 (0.426–2.057)	0.962 (0.447–2.166)	.931
Model 2	1.000	0.953 (0.449–2.157)	0.779 (0.353–1.840)	0.977 (0.436–1.258)	.948
Model 3	1.000	1.234 (0.478–3.231)	0.794 (0.291–2.299)	1.176 (0.398–3.352)	.805
Whole grains–plant protein	≤−0.617	(−0.617, −0.276)	(−0.276, 0.268)	>0.268	–
Model 1	1.000	0.870 (0.414–1.714)	0.535 (0.227–1.167)	0.492 (0.219–1.154)	.320
Model 2	1.000	0.817 (0.335–1.908)	0.505 (0.206–1.350)	0.486 (0.166–1.254)	.256
Model 3	1.000	0.805 (0.330–1.899)	0.501 (0.200–1.364)	0.472 (0.164–1.243)	.359
Nuts–fruits	≤−0.485	(−0.485, −0.243)	(−0.243, 0.238)	>0.238	–
Model 1	1.000	1.017 (0.468–2.154)	1.242 (0.592–2.530)	0.575 (0.235–1.346)	.359
Model 2	1.000	0.938 (0.416–2.079)	1.190 (0.551–2.540)	0.526 (0.224–1.310)	.345
Model 3	1.000	1.094 (0.432–2.890)	1.451 (0.567–3.670)	0.746 (0.248–1.227)	.620
Refined grains–vegetables	≤−0.715	(−0.715, −0.120)	(−0.120, 0.552)	>0.552	–
Model 1	1.000	0.609 (0.268–1.368)	0.810 (0.376–1.702)	0.859 (0.468–1.786)	.706
Model 2	1.000	0.531 (0.235–1.210)	0.739 (0.349–1.578)	0.848 (0.399–1.857)	.287
Model 3	1.000	0.482 (0.180–1.309)	0.672 (0.266–1.715)	1.201 (0.475–3.037)	.302
Dairy	≤−0.680	(−0.680, −0.241)	(−0.241, 0.634)	>0.634	–
Model 1	1.000	0.832 (0.378–1.860)	0.933 (0.436–2.018)	1.007 (0.470–2.140)	.884
Model 2	1.000	0.759 (0.328–1.735)	0.973 (0.436–2.209)	1.024 (0.462–2.271)	.513
Model 3	1.000	0.826 (0.181–1.284)	1.071 (0.405–2.787)	1.011 (0.379–2.533)	.403
Sugary beverages	≤−0.247	(−0.247, −0.181)	(−0.181, −0.095)	>−0.095	–
Model 1	1.000	1.104 (0.562–5.670)	1.131 (0.434–2.948)	2.166 (0.902–5.118)	.020
Model 2	1.000	2.183 (0.884–3.544)	1.072 (0.307–2.494)	1.796 (0.742–4.472)	.432
Model 3	1.000	5.333 (1.795–16.129)	1.647 (0.475–5.594)	3.710 (1.242–10.872)	.021

Model 1: Unadjusted for confounding factors.

Model 2: Adjusted for other dietary patterns.

Model 3: Adjusted for age, duration of diabetes, region, education level, occupation, annual per capita household income, annual per capita household expenditure, smoking, alcohol consumption, weekly physical activity level, exercise intensity, daily sleep duration, body mass index (BMI), waist circumference, systolic blood pressure, diastolic blood pressure, fasting plasma glucose, glycated hemoglobin (HbA1c), total cholesterol, triglycerides, low-density lipoprotein cholesterol (LDL-C), high-density lipoprotein cholesterol (HDL-C), and serum uric acid.

HbA1c = glycated hemoglobin, HDL-C = high-density lipoprotein cholesterol, LDL-C = low-density lipoprotein cholesterol.

## 4. Discussion

The impact of dietary patterns on glycemic control is a critical area of research in the management of T2DM. In community-managed settings, where individualized care may be limited, understanding how different dietary habits influence glucose regulation is essential for developing effective, population-based dietary interventions. Previous studies have shown that diets rich in plant-based foods, fiber, and healthy fats are associated with better glycemic outcomes, while diets high in refined carbohydrates and added sugars tend to impair glucose metabolism.^[13-15]^ This study provides valuable insights into the comparative effects of different dietary patterns on glycemic control in community-managed patients with T2DM. By employing factor analysis, we identified 6 distinct dietary patterns that reflect the dietary habits of this population. The subsequent analysis of their associations with FPG and postprandial 2-hour blood glucose control revealed significant findings that contribute to a better understanding of the dietary factors influencing glycemic outcomes in T2DM management.

Our results indicate that the nuts–fruits dietary pattern, characterized by high consumption of nuts and fresh fruits, was associated with a significantly reduced risk of poor FPG control. Specifically, patients in Q4 of this pattern had a 49.9% lower risk of inadequate FPG control compared to those in Q1. This finding underscores the potential protective role of a diet rich in healthy fats, fiber, and micronutrients – nutrients that are abundant in nuts and fruits. The beneficial effects of this pattern on glycemic control could be attributed to several mechanisms. Nuts are rich in unsaturated fatty acids, which have been shown to improve insulin sensitivity and reduce inflammation, both of which are critical in managing glucose levels.^[16,17]^ Moreover, the high fiber content in both nuts and fruits can slow the absorption of carbohydrates, resulting in more stable postprandial glucose levels. Fruits, particularly those with low glycemic indices, provide essential vitamins, minerals, and antioxidants, which may also support better metabolic function and reduce oxidative stress, a known contributor to insulin resistance.^[18,19]^ These combined effects likely contribute to the observed reduction in poor glycemic control among patients adhering to this dietary pattern.

In contrast, the sugary beverages dietary pattern was strongly associated with a higher risk of poor postprandial 2-hour blood glucose control. Patients in Q4 of this pattern had a 3.710-fold increased risk of poor postprandial glucose regulation compared to those in Q1. This result highlights the detrimental impact of consuming high amounts of sugary beverages, which includes fruit and vegetable juices, as well as beverages with added sugars. The mechanisms underlying this relationship are well-established. Sugary beverages are rapidly absorbed, leading to a sharp increase in blood glucose levels and a subsequent spike in insulin secretion. Regular consumption of such beverages can result in chronic hyperglycemia, which over time impairs the body’s ability to regulate glucose effectively.^[20,21]^ Additionally, the frequent intake of sugary drinks is associated with increased calorie consumption and weight gain, both of which exacerbate insulin resistance, a key factor in poor glycemic control in T2DM patients. These findings align with previous research demonstrating that high sugar intake, particularly from beverages, is a significant risk factor for the development of metabolic disorders, including T2DM. Given the well-documented harmful effects of sugary beverages, reducing their intake should be a central focus of dietary interventions aimed at improving postprandial glycemic control in patients with T2DM.^[22,23]^

The dietary patterns identified in this study reflect a diverse range of food group combinations, each of which plays a distinct role in glycemic management. The animal protein pattern, characterized by high intakes of livestock meat, poultry, and seafood, may provide benefits through high-quality protein intake, although its impact on glycemic control in this study was less pronounced compared to other patterns. Proteins are known to have a minimal effect on postprandial glucose levels; however, excessive consumption of red and processed meats has been associated with increased insulin resistance, possibly counteracting potential benefits.^[24,25]^ The whole grains–plant protein and refined grains–vegetables patterns reflect a balance of complex carbohydrates and dietary fiber, both of which have been shown to improve glycemic control by slowing the absorption of glucose. These patterns emphasize the importance of fiber-rich plant foods, which are essential for maintaining stable blood glucose levels and reducing insulin spikes. In contrast, the Dairy pattern, which was dominated by dairy products, showed minimal association with FPG or postprandial glucose control in this study.^[26,27]^ While dairy products are a good source of calcium and protein, their role in glycemic management remains debated, with some studies suggesting that high-fat dairy may impair insulin sensitivity, while others point to potential benefits from low-fat dairy products.

### 4.1. Strengths and limitations

This study also presents several notable strengths. First, it utilizes a relatively large sample of 860 community-managed T2DM patients, enhancing the statistical power and reliability of the findings. Second, dietary patterns were identified using factor analysis, a data-driven approach that reflects real-world dietary behaviors rather than predefined dietary models. Third, the use of a culturally adapted food frequency questionnaire and comprehensive adjustment for a wide range of sociodemographic and clinical confounders strengthens the internal validity of the results. Moreover, the separate analysis of fasting and postprandial glycemic outcomes offers nuanced insights into the specific impact of dietary components on different aspects of glycemic control. However, this study has several limitations. First, the cross-sectional design limits the ability to establish causal relationships between dietary patterns and glycemic control. Longitudinal studies or randomized controlled trials are needed to confirm these associations. Second, dietary intake was self-reported, which may introduce recall bias and inaccuracies in estimating food consumption. Additionally, the study was conducted in a specific community-managed population, which may limit the generalizability of the findings to other populations with different cultural or dietary habits. Future research should focus on conducting prospective studies to explore the long-term effects of these dietary patterns on glycemic outcomes. Moreover, investigations into the mechanisms underlying the relationship between specific food groups and glucose metabolism could provide deeper insights into optimizing dietary recommendations for T2DM patients. Expanding the study to diverse populations will also be crucial in validating the findings and improving global diabetes management strategies.

## 5. Conclusions

This study highlights the significant influence of dietary patterns on glycemic control in community-managed type 2 Diabetes patients. The nuts–fruits pattern was associated with improved FPG control, while the sugary beverages pattern was linked to poor postprandial glucose regulation. These findings underscore the importance of promoting healthy, nutrient-rich diets for better diabetes management.

## Acknowledgments

We extend our heartfelt thanks to all the participants and colleagues who contributed to this study.

## Author contributions

**Conceptualization:** Yanling Zheng, Haiyan Fan, Wen-Yong Knog.

**Data curation:** Yanling Zheng, Haiyan Fan.

**Formal analysis:** Yanling Zheng.

**Investigation:** Yanling Zheng, Haiyan Fan.

**Methodology:** Yanling Zheng, Haiyan Fan, Wen-Yong Knog.

**Resources:** Yanling Zheng, Haiyan Fan.

**Software:** Yanling Zheng.

**Writing – original draft:** Yanling Zheng, Haiyan Fan.

**Writing – review & editing:** Wen-Yong Knog.
